# Recent Progress in Cellulose Hydrophobization by Gaseous Plasma Treatments

**DOI:** 10.3390/polym16060789

**Published:** 2024-03-12

**Authors:** Gregor Primc, Alenka Vesel, Rok Zaplotnik, Marija Gorjanc, Peter Gselman, Marián Lehocký, Miran Mozetič

**Affiliations:** 1 Department of Surface Engineering, Jozef Stefan Institute, Jamova Cesta 39, 1000 Ljubljana, Slovenia; alenka.vesel@ijs.si (A.V.); rok.zaplotnik@ijs.si (R.Z.); miran.mozetic@ijs.si (M.M.); 2 Faculty of Natural Sciences and Engineering, University of Ljubljana, Aškerčeva 12, 1000 Ljubljana, Slovenia; marija.gorjanc@ntf.uni-lj.si; 3 Interkorn Ltd., Gančani 94, 9231 Beltinci, Slovenia; peter.gselman@interkorn.si; 4 Centre of Polymer Systems, University Institute, Tomas Bata University in Zlín, Trida Tomase Bati 5678, 760 01 Zlin, Czech Republic; lehocky@post.cz

**Keywords:** cellulose, hydrophobization, gaseous plasma, PTFE, PDMSO

## Abstract

Cellulose is an abundant natural polymer and is thus promising for enforcing biobased plastics. A broader application of cellulose fibers as a filler in polymer composites is limited because of their hydrophilicity and hygroscopicity. The recent scientific literature on plasma methods for the hydrophobization of cellulose materials is reviewed and critically evaluated. All authors focused on the application of plasmas sustained in fluorine or silicon-containing gases, particularly tetrafluoromethane, and hexamethyldisiloxane. The cellulose materials should be pre-treated with another plasma (typically oxygen) for better adhesion of the silicon-containing hydrophobic coating. In contrast, deposition of fluorine-containing coatings does not require pre-treatment, which is explained by mild etching of the cellulose upon treatment with F atoms and ions. The discrepancy between the results reported by different authors is explained by details in the gas phase and surface kinetics, including the heating of samples due to exothermic surface reactions, desorption of water vapor, competition between etching and deposition, the influence of plasma radiation, and formation of dusty plasma. Scientific and technological challenges are highlighted, and the directions for further research are provided.

## 1. Introduction

**Figure 1:**
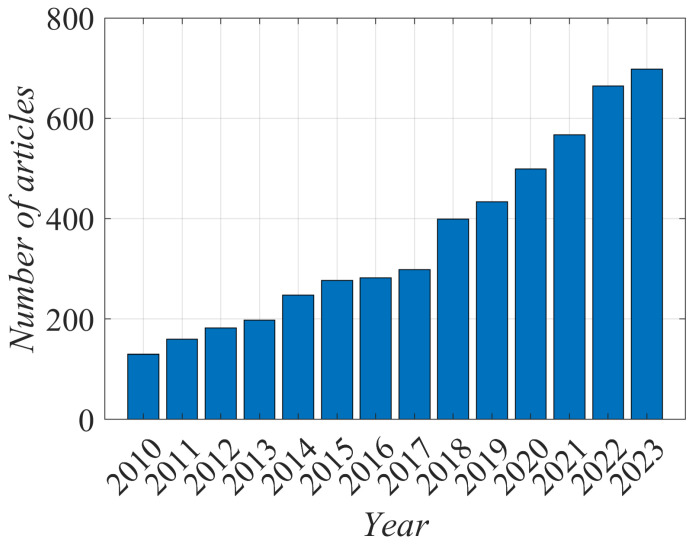
The number of scientific articles on hydrophobization of cellulose (retrieved from the Web of Science).

Cellulose particles of various sizes and morphology are among the most promising biobased materials for the reinforcement of polymer-matrix composites [1,2]. The drawback, however, is inadequate adhesion between the polymer matrix and the cellulose fillers due to a mismatch in surface energies. Most polymers are at least moderately hydrophobic, and cellulose is a hydrophilic material. The hydrophilicity is due to the cellulose composition and structure. The mismatch could be suppressed by hydrophobization, i.e., decreasing the surface energy of the cellulose materials. Cellulose hydrophobization has attracted enormous attention from the scientific community as well as users. Several review articles have been published, and a recent one is [3]. The number of scientific papers focused on the hydrophobization of cellulose materials is still increasing despite the tremendous efforts to develop methods for rapid, stable, and ecologically acceptable modification of cellulose surface properties, which should lead to the appropriate hydrophobic character of treated samples. Figure 1 represents the number of scientific papers published annually since 2010.

A standard method for tailoring surface properties of polymers is the application of non-equilibrium gaseous plasma [4,5,6]. Gaseous plasma is a source of charged particles and molecular radicals [7] as well as photons with an energy that exceeds the binding energy of atoms in the polymer materials [8]. The energetic photons break bonds in the surface film of all polymers, including the very strong C−F bonds in fluorinated polymers [9]. The treatment uniformity in large plasma reactors may be questionable because of significant gradients in the density or reactive plasma species [10]. Furthermore, the gaseous impurities may influence the properties of gaseous plasma so that the surface finish may deviate significantly from the desired degree of hydrophilicity. The source of gaseous impurities may be the desorption of various molecules (usually water vapor) from the substrates themselves, and this is likely to occur in the case of hygroscopic materials such as cellulose [11].

Depending on the parameters, gaseous plasma may cause functionalization, etching, deposition of a coating, and/or modification of the subsurface film. Functionalization stands for the irreversible bonding of plasma species onto the polymer surface and is frequently used for the hydrophilization of polymers [12]. Precise dosing of plasma radicals enables the functionalization of polymers with desired functional groups [13]. Etching is often a consequence of synergy between neutral radicals and bombardment with energetic ions from gaseous plasma [7] but also occurs upon treatment with neutral radicals only [7]. Plasma containing condensable radicals will cause the formation of a thin surface film with a composition related to the type of radicals in gaseous plasma. The thin films could be either inorganic or polymer-like, and the technique used to synthesize thin polymer films on substrates is called plasma polymerization [14]. Among the most studied hydrophobic films deposited by plasma polymerization are those resembling polydimethylsiloxane [15]. Finally, plasma treatment also causes modification of the sub-surface film, usually due to the absorption of energetic photons. Namely, most plasmas are extensive sources of vacuum ultraviolet (VUV) radiation, i.e., radiation in a range of wavelengths between about 100 and 200 nm, corresponding to the photon energy between 12 and 6 eV, respectively. The VUV photons are absorbed in a surface film of a thickness of the order of between 10 and 100 nm and break bonds in the polymer materials. The effect is irreversible if the bonds are not re-established after absorbing the VUV photons. Hence, the absorption of VUV radiation causes structural modifications of the surface film of the thickness, which corresponds to the penetration depth of the VUV photons.

## 2. Cellulose Hydrophobization by Gaseous Plasma Treatment

Hydrophobization of cellulose materials by gaseous plasma treatments has attracted significant attention from the scientific community. Functionalization has rarely been tackled, and deposition of thin films by plasma polymerization seems to be preferred. Different research groups used various discharges for sustaining gaseous plasma. Traditionally, the plasma hydrophobization of polymers has been accomplished in low-pressure plasma reactors, but many authors also utilized plasmas sustained at atmospheric pressure. The intermediate range of pressures, i.e., between a few mbar and 1 bar, has yet to be tackled. The authors used various gases or gas mixtures and different discharges to sustain gaseous plasma appropriate for the hydrophobization of cellulose materials. The recent progress in this scientific niche is presented, and the results reported by various authors are critically assessed. Only the scientific literature published in the last 5 years (after 2018) is considered in this review.

### 2.1. Deposition of PDMSO-like Coatings

Polydimethylsiloxane is renowned for its hydrophobicity, so numerous authors reported increased hydrophobicity of cellulose after treating it with plasma, which enables the deposition of such coatings. Yang et al. [16] reported a super-hydrophobic surface finish of cotton fabrics after treatment with atmospheric pressure plasma powered by a radio-frequency (RF) discharge operating at 18 kHz. The cotton fabrics were mounted 4 cm below the plasma nozzle to prevent overheating of the samples during treatment. They used either oxygen or nitrogen and added hexamethyldisiloxane (HMDSO) vapor mixed with argon into the discharge zone. The plasma treatment resulted in 150° and 160° water contact angles when the carrier gases were nitrogen and oxygen, respectively. The authors have not reported the treatment time but rather the speed of the plasma jet moving over the cotton fabrics, which was fixed at 5 m/min with a raster offset of 2 mm. Considering these data, the treatment time can be estimated to be approximately 0.1 s. Plasma treatment caused the deposition of nanoparticles containing oxygen, silicon, and probably carbon and hydrogen. The deposition of nanoparticles is explained by the formation of dusty plasma. The HMDSO precursor dissociates partially upon plasma conditions, and the radicals tend to form negatively charged clusters in the gaseous plasma. The clusters grow by condensation of radicals on their surface and finally form nanoparticles, which are deposited on the cotton substrate. The nanoparticles are hydrophobic, and the rich morphology adds to the water contact angle, thus making the fabrics almost super-hydrophobic. The effect was permanent, and the nanoparticles persisted on the surface of cotton fibers also after washing.

Cerny et al. [17] treated cotton fabrics with a plasma sustained by an atmospheric-pressure gliding arc operating at a peak voltage of 10 kV, a frequency of 50 Hz, and an input power of 750 W. Plasma was sustained in the air with an admixture of HMDSO. The non-treated samples were highly hydrophilic. A water droplet was absorbed in a fraction of a second, so the authors could not measure the contact angle of untreated cellulose. The samples were placed several cm away from the glowing plasma and treated for several minutes. The water contact angle (WCA) on many samples was about 140° after the treatment. No absorption of a water droplet was observed for some samples because the water droplet evaporated instead after storing the samples for prolonged times. The distance between the glowing plasma and the cellulose samples of 8 cm, the gas flow rate of 14 slm, and the treatment time of 30 min were found particularly beneficial for stable hydrophobization of the cotton samples. Scanning electron microscope (SEM) analyses revealed the deposition of nanoparticles on the textile fibers, while Fourier-transform infrared spectroscopy (FTIR) revealed a composition resembling polydimethylsiloxane with excessive SiO_x_ compound. Washing, however, caused a partial release of the nanoparticles from the fabric surface, so a water droplet deposited onto the washed samples was slowly absorbed into the textile. The residence time of the water droplet was about 2 min after the first washing and about a minute after the third washing.

**Figure 2:**
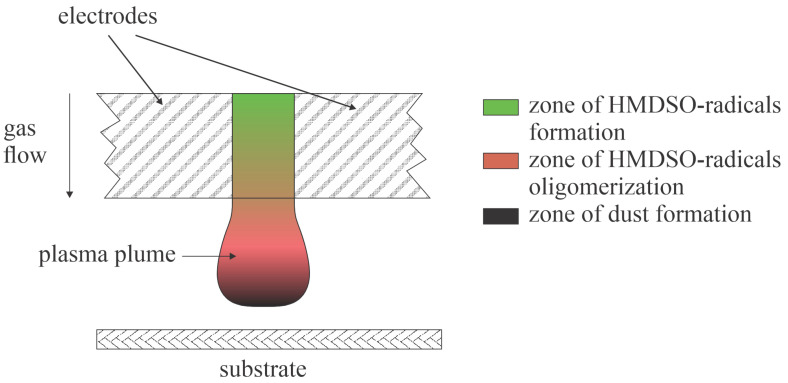
Illustration of HMDSO-radicals kinetics in atmospheric pressure plasmas.

The gas-phase reactions in the atmospheric-pressure methods used for the deposition of hydrophobic coatings by Yang et al. [16] and Cerny et al. [17] are illustrated in Figure 2. The gaseous precursors enter the gaseous plasma, which is sustained by an electric field between the electrodes. The plasma plume is not limited to the volume between the electrodes but expands towards the cellulose substrates because of the gas flow and the peculiarities of the discharges (plasma jet in [16] and gliding arc [17]). The HMDSO molecules entering the plasma are radicalized by electron-impact dissociation, and the intensity of radical formation is the highest in the region of the highest electric field, i.e., between the electrodes. Once the radicals become dense enough to start colliding with each other, the oligomerization is triggered. The oligomerization proceeds along the gas flow and the oligomers start forming particles, which represent condensation seeds for adhesion of more HMDSO-radicals. The particles grow in size along the gas flow and finally rich the cellulose substrates where they adhere. The effect is illustrated in Figure 2.

**Figure 3:**
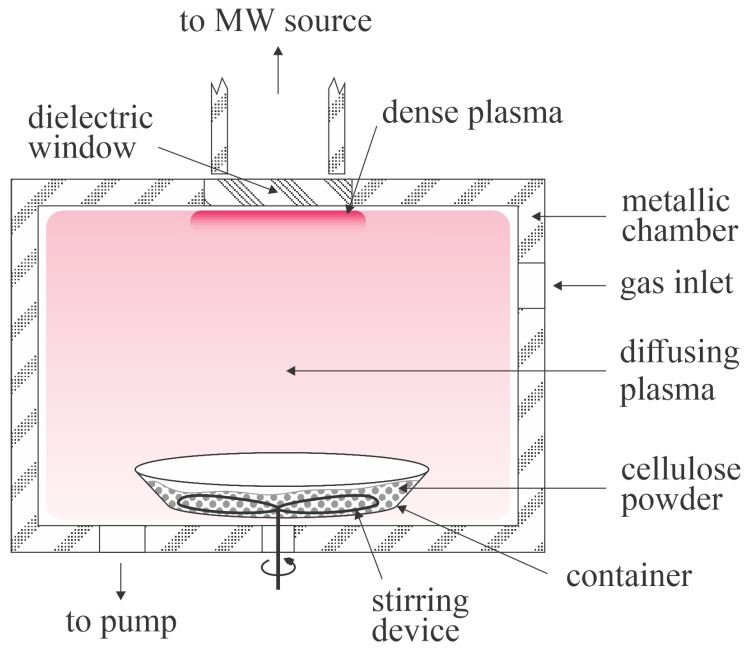
Schematic of the low-pressure plasma reactor for cellulose hydrophobization used by Cerny et al. [18].

In another paper [18], the same group as [17] used a completely different experimental setup to obtain highly hydrophobic organosilane-functionalized cellulose. They used a low-pressure plasma reactor powered by a microwave discharge operating at the power of 500 W. The cellulose fibers with an average length/width of 65 µm/33 µm were placed into a dish equipped with a stirring device. They were first treated with argon, oxygen, or air plasma sustained at the pressure of 100 Pa and the plasma treatment time varied between 15 and 90 min. After this plasma treatment, the experimental chamber was evacuated, and HMDSO vapors were leaked into the plasma reactor at the flow rate of 5 sccm during continuous pumping. No significant agglomeration occurred in the gas phase due to the low-pressure conditions. The pre-treatment with argon plasma did not enable significant hydrophobization of the fibers after plasma polymerization. However, the pre-treatments with air and oxygen plasma enabled gradual hydrophobization with increasing treatment time, so the final WCA after 90 min of plasma treatment was 139° and 143°, respectively. The difference between argon and oxygen or nitrogen is in the formation of O or N atoms which activate the polymer surface. The oxygen plasma pre-treatment caused a faster hydrophobization. The experimental setup used by Cerny et al. [18] is schematically presented in Figure 3. The gas inlet and pump duct position prevented most HMDSO molecules from reaching the zone of intensive plasma, which was limited to the volume next to the waveguide, as shown in Figure 3. The zone is limited to a small volume due to the small penetration depth of microwaves in a conductive medium, i.e., dense plasma. The HMDSO molecules are therefore subjected practically only to the weak diffusing plasma which occupied the entire volume of the discharge chamber. The formation of HMDSO radicals in the weak plasma is poor, which explains the long treatment times needed to hydrophobize the cellulose surface. The pre-treatment with air or oxygen plasma was essential for binding poorly radicalized HMDSO precursor. Oxygen plasma caused the formation of more polar groups on the cellulose materials than air plasma, which explains the shorter HMDSO-plasma treatment time to achieve the desired surface finish.

Matouk et al. [19] exposed cellulose nanocrystalline films to atmospheric pressure plasma sustained in a mixture of argon and silane (0.02 vol.%) to create the super-hydrophobic surface finish. Plasma was sustained by a standard parallel-plate dielectric barrier discharge (DBD) discharge powered by an AC generator operating at the peak voltage of 6 kV and the frequency of 5.5 kHz. The treatment times were between 1 and 3 min. The water contact angle on the as-synthesized cellulose nanocrystalline films was 53° and increased with increasing plasma treatment time. The WCA was 60°, 70°, and 150° after treating the films for 1, 2, and 3 min, respectively. The nonlinearity of the WCA versus the treatment time was explained by different chemistry of deposited silicon, which was studied by XPS. While a 1-min treatment caused the predominant formation of Si−Si and Si−H bonds on the sample surface, prolonged treatment favored the formation of SiO_2_ bonds. The relative concentration of Si, as deduced from survey XPS spectra, remained constant at about 60 at.% for all deposition times. The authors attributed the oxidation of silicon to the water vapor, which slowly desorbed from the nanocellulose films. The low concentration of silane in argon definitely suppresses the formation of agglomerates in the gas phase, as illustrated in Figure 2.

Leal et al. [20] used tri-chloromethyl silane (TCMS) to deposit hydrophobic coatings on membranes synthesized from bacterial cellulose. The membranes were first treated with oxygen plasma sustained by a radiofrequency discharge which operated at the frequency of 40 MHz and the power of 100 W. A low-pressure reactor was used, and the oxygen pressure during treatment was 100 Pa. The oxygen-plasma treatment resulted in nanostructuring and activation of the membrane surface, which was helpful for the adhesion of the hydrophobic coating. The coating was deposited by the standard (without plasma) chemical vapor deposition (CVD) technique. The oxygen plasma-treated membranes were exposed to the TCMS vapors for an hour at 95 °C. The oxygen plasma treatment alone did not cause a measurable modification of the membrane wettability since the water contact angle remained at about 25°. After depositing the coating, however, the membrane assumed the WCA as large as 133°. The hydrophobic character of the membranes also persisted after prolonged storage in distilled water, but the WCA slowly decreased with increasing storage time. The effect of oxygen plasma pre-treatment was therefore beneficial, the same as reported by Cerny et al. [18].

Yao et al. [21] fabricated highly hydrophobic cellulose films by treating cellulose fibers with oxygen plasma, followed by the condensation reaction with TriSilanollsobutyl-Polyhedral oligomeric silsesquioxane (TS-POSS). The final water contact angle was as large as 153°. The oxygen plasma treatment of the cellulose fibers was performed to activate the hydroxyl groups on the surface of the fibers. Plasma-treated fibers were dipped into the TS-POSS solution, and a thin hydrophobic coating was formed by the condensation reaction between the cellulose and TS-POSS hydroxyl groups. The materials were treated again with oxygen plasma for 500 s to etch the TS-POSS. The oxygen pressure was 25 Pa, and the discharge power was 100 W. According to the authors [21], the amorphous areas were etched preferentially, so nanostructured surfaces were obtained. Without treatment with oxygen plasma, the materials exhibited only a moderate WCA of 107°. 

Babaei et al. [22] reported plasma deposition of hydrophobic coatings on porous cellulosic substrates using a standard DBD discharge operating at 20 kHz to sustain the plasma in a mixture of helium and HMDSO at atmospheric pressure. The treatment time was 30 min. The He flow rate was 4500 sccm, and HMDSO was admixed at 120 mg/h. The substrates were porous hand sheets made of bleached, unrefined softwood Kraft pulp. The treatment caused the static water contact angle of 140–150°, and the water adsorption time was immeasurably long (over 300 s). The gas phase kinetics in the experimental setup used by Babaei et al. [22] were similar to that illustrated in Figure 2.

Table 1 summarizes process parameters, such as pressure, power, time, and gas, used for the deposition of PDMSO-like coatings for hydrophobization purposes, and the final water contact angle.

### 2.2. Deposition of Fluorine-Containing Coatings

Fluorinated polymers are renowned for their hydrophobic character, so surface fluorination is a natural choice for the hydrophobization of any polymer, including cellulose. Samanta et al. [23] used atmospheric-pressure plasma sustained in helium with an admixture of tetrafluoroethane (TFE) to treat viscose fabrics. The DBD discharge was sustained with an RF generator operating at the voltage of 6 kV and frequency of 17.4 kHz. The gas flow rates of He and TFE varied between 300–1700 and 5–500 sccm, respectively, and the treatment time was 1–8 min. The WCA on non-treated samples was immeasurably low, but even a 1-min plasma treatment caused the WCA to be 127°. The WCA increased with increasing treatment time and reached 153° after treating the viscose fabrics for 8 min. High-resolution XPS C1s spectra revealed the appearance of CF_x_ functional groups on the cellulose substrates. The main peak was attributed to C-C and C-H bonds rather than C-O, and it persisted even after treating the cellulose samples for 8 min. The high WCA was explained as a combined effect of sample morphology and coating with a thin film resembling polytetrafluoroethylene.

Oberlintner et al. [24] treated films synthesized from nanocellulose fibers. They used a low-pressure plasma reactor to deposit a thin film of fluorinated carbon onto the cellulose surface by plasma polymerization. The plasma reactor was a glass tube with a diameter of 4 cm. Plasma was sustained in tetrafluoromethane (CF_4_) with inductively coupled RF discharge powered by an RF generator, which operated at the standard frequency of 13.56 MHz and output power of 80 W. The CF_4_ flow rate of 80 sccm was reported, so the pressure should be of the order of 10 Pa. The WCA gradually increased from the initial 46° to about 90° after 10 s and 130° after 55 s of plasma treatment. The major peak in the high-resolution C1s XPS spectrum for untreated samples was attributed to the C-O bond, and its relative intensity decreased with increasing plasma treatment time. XPS also confirmed the presence of C-F_x_ bonds on the surface of treated cellulose.

In another paper, Oberlintner et al. [25] used the same discharge tube for sustaining plasma in CF_4_. Unlike most other authors, they also reported the ultimate pressure achieved in the vacuum system, which was 1 Pa. In this paper, the authors chose the output power of the RF generator of 150 W, but the samples were placed 7 cm away from the RF coil, where the diffusing plasma was sustained. The CF_4_ pressure was 50 Pa, and the pumping speed of a rotary vacuum pump was 80 m^3^/h. The samples were chitosan/cellulose nanocrystals composite in the form of thin films. The plasma treatment time varied between 0.5 and 30 s. The WCA of untreated samples was 105° and increased to about 120° even after 1 s of plasma treatment. Prolonged treatment caused a slow increase in the WCA, which assumed 127° after treating the films for 30 s. The hydrophobic surface finish was found to be permanent since the WCA did not change significantly even after a month of aging. In this paper, Oberlintner et al. [25] reported the overlapping of the peaks corresponding to C-O and C-C-F which makes the interpretation of the high-resolution C1s spectrum complicated. 

Kawano et al. [26] also utilized CF_4_ plasma to coat cellulose with a thin film of fluorinated carbon. They used regenerated cellulose to synthesize thin films. A cellulose film was then exposed to plasma sustained in a vacuum chamber at the pressure of 67 Pa with a capacitively coupled RF generator. The discharge power was varied from 10 to 80 W. The discharge power of 40 W enabled the highest degree of hydrophobization. The initial WCA was 40° and increased to about 115° after treatment with CF_4_ plasma for 30 s and 120° after 60 s of treatment. After that, however, the WCA decreased with further plasma treatment and dropped down to 25° after 240 s of treatment. The hydrophobization was explained by etching with ions and functionalization with fluorine-containing functional groups. The authors explained the decreasing WCA with prolonged treatment time by the saturation of the chemical bond between fluorine and cellulose, as well as an increased number of defects. XPS revealed increased fluorine concentration after treatment for 60 s and much smaller after treatment for 240 s.

Ioannou et al. [27] reported a super-hydrophobic surface finish of cellulose acetate membranes. The membranes were first treated with low-pressure oxygen plasma sustained by a capacitively coupled RF discharge. The oxygen plasma caused controlled etching and surface activation. After treating the membranes with oxygen plasma, the samples were transferred to another system, where the plasma was sustained by an inductively coupled RF generator operating at a power as high as 900 W. Octafluorocyclobutane was introduced at the flow rate of 25 sccm during continuous pumping, so the C_4_F_8_ pressure was 5.33 Pa. The C_4_F_8_ plasma treatment time of 4 min enabled the WCA above 160°. SEM images revealed rich morphology, which was explained by etching of the membranes during the first treatment with oxygen plasma. The morphology was preserved during the deposition of a thin C_x_F_y_ film by plasma polymerization.

**Figure 4:**
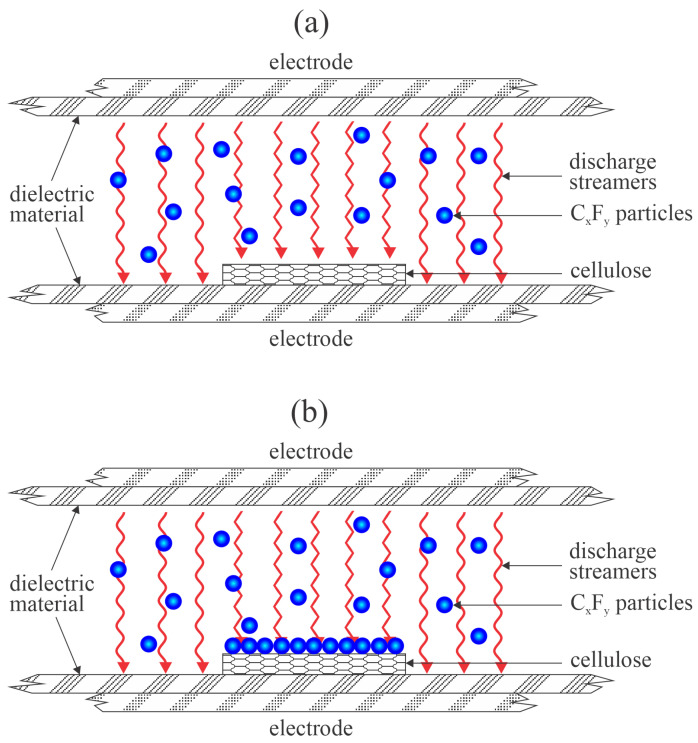
Illustration of the Teflon-like nanoparticle deposition when using atmospheric pressure plasma sustained by a dielectric barrier discharge in F-containing precursor. (**a**) Soon after turning on the discharge, (**b**) after prolonged treatment.

The interaction between plasma sustained in fluorine-containing gases and cellulose materials is complex and still inadequately understood. Namely, unlike the HMDSO or similar precursors, fluorine-containing gases will produce a significant concentration of F atoms upon plasma conditions, and the fluorine atoms will etch many materials, including the cellulose samples and the chamber walls if the chamber is made of glass. At the atmospheric pressure, the F-containing precursor will partially dissociate, and the resultant radicals will either interact with an F atom or between each other, thus forming the radical dimers and oligomers, which will stick together in the gas phase and form C_x_F_y_ nanoparticles. The nanoparticles will adhere to the surfaces and cause the formation of a film of rich morphology and Teflon-like composition, thus making the substrate super-hydrophobic, as disclosed by Samanta et al. [23]. The treatment of cellulose materials with a fluorine-containing plasma sustained by a DBD discharge at atmospheric pressure is illustrated in Figure 4. 

**Figure 5:**
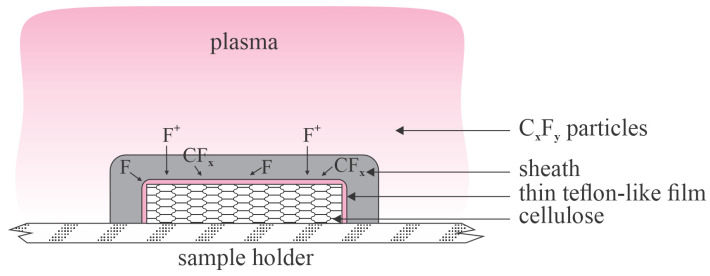
Interaction of a low-pressure plasma sustained in a fluorocarbon gas with cellulose materials. The F^+^ ions are accelerated perpendicularly to the cellulose surface and bombard it with a significant kinetic energy. The flux of neutral species (CF_x_ and F radicals) is random. VUV radiation (not shown in the illustration) adds to the complexity of plasma-cellulose interaction.

The illustration of the interaction between a fluorine-containing plasma and the cellulose material under low-pressure conditions is more complex. While some oligomerization in the gas phase occurs even at the pressure of about 1 mbar [28], the low collision frequency suppresses the formation of the C_x_F_y_ nanoparticles, so the deposited films are smoother. This fact explains moderate water contact angles reported by authors who used low-pressure plasmas for the hydrophilization of cellulose materials (see Table 2). Low-pressure plasmas sustained in fluorine-containing precursors contain a significant concentration of F^+^ ions. Fluorine is an electronegative gas, so there is a large concentration of F^−^ ions in the plasma. The negative charge is compensated by F^+^ (as well as CF_x_^+^) ions. The F^+^ ions accelerate in the sheath next to the plasma-facing material and acquire a significant kinetic energy. The F^+^ ions will etch the material-facing plasma, so the net effect of the plasma-substrate interaction is a competition between the deposition of CF_x_ radicals and the etching of deposited films. Furthermore, although not as reactive as ions, the F atoms are also likely to etch the deposited films, as explained in the classical paper by Booth [28]. The balance between etching and deposition depends significantly on the concentration of gaseous impurities, as well as the discharge power [29]. Adding an F-scavenger (the best is H atoms) into the plasma will ensure predominant deposition, but a hydrogen admixture has not been adopted by authors whose results are summarized in Table 2 despite it having been known for decades [29]. On the contrary, the addition of oxygen and/or water vapor will favor etching [11]. The interaction of low-pressure plasma sustained in the fluorine-containing precursor with a cellulose sample is illustrated in Figure 5. 

### 2.3. Deposition of Hydrocarbon Coatings

Matouk et al. [19] also exposed cellulose nanocrystalline films to plasma sustained in a mixture of argon and methane. The concentration of CH_4_ in Ar was 2 vol.%, and plasma treatment was performed at atmospheric pressure. The WCA slowly increased with increasing treatment time and stabilized at 100° after treating the samples for 18 min. The concentration of the C−C/C−H peak, as deduced from high-resolution XPS C1s peaks, followed the behavior of the WCA. The concentration of C=O/O−C−O peak dropped from the original 17 to 11 at.% after a minute of plasma treatment and dropped even further, below the detection limit, for treatment times 8 min and above. Interestingly, the C−O groups persisted even for the longest treatment time of 30 min. The authors explained the improved hydrophobicity by the formation of a polyolefin-like coating on the cellulose nanocrystal films. The CH_4_ molecules were transformed to CH_x_ radicals upon plasma conditions, and the radicals were stacked on the substrates. Formation of C_x_H_y_ particles in the gas phase (Figure 2) might have occurred as well, and the particles might have adhered to the substrates, although the atomic force microscope (AFM) images did not reveal any modification of the topography, at least not on the 5 µm × 5 µm scale.

## 3. Discussion

The survey of recent literature reveals that the deposition of either silicon- or fluorine-containing thin films remains a hot topic of hydrophobization of cellulose materials. The recent results do not deviate much from early reports. For example, Zanini et al. elaborated on the influence of thin hydrophobic films prepared by plasma polymerization using HMDSO as a precursor on the evolution of surface wettability of various polymer materials [30]. Mahlberg reported a hydrophobic surface finish of lignocellulosic materials after treatment with HMDSO plasma [31]. Inagaki et al. used various mixtures of CF_4_ with hydrocarbons to synthesize highly hydrophobic films by plasma polymerization [32]. Hochart et al. [33] probed several fluorocarbons as precursors in plasma polymerization and found rapid deposition of polytetrafluoroethylene-like films, which caused a significant decrease in the surface free energy. The critical scientific challenge remains the description of the surface kinetics upon hydrophobization of cellulose materials, and the technological challenge is the invention of methods for rapid hydrophobization on an industrial scale.

### 3.1. Hydrophobization versus Precursor Pressure and Plasma Treatment Time

Plasma could be sustained at any pressure between less than 1 Pa and several bars. As revealed in Table 1 and Table 2, the recent authors used either the low-pressure range between 5.33 and 67 Pa or the atmospheric pressure (10^5^ Pa). There is a significant difference in the plasma physics sustained in the low-pressure range and at the atmospheric pressure. Namely, the plasma properties are governed predominantly by the surface effects at low pressure [34] and the gas-phase reactions at atmospheric pressure [35].

Low-pressure hydrophobization of cellulose materials is usually performed by dosing pure precursors into the vacuum system. The atmospheric-pressure hydrophobization, however, is performed by using a carrier gas. There are several reasons for using a carrier gas. The trivial one is the natural limitation of the saturated vapor pressure. HMDSO’s saturated vapor pressure is 100 mbar at 310 K and 1 bar at 373 K [36]. Pure HMDSO at atmospheric pressure will condense spontaneously at temperatures below 373 K, so either the experimental system must be heated to prevent this effect, or a carrier gas should be used to keep the partial pressure of HMDSO below the saturated vapor pressure at a given temperature. This limitation is obsolete for CF_4_ because its vapor pressure is 1 bar at a temperature just below 150 K, so well below room temperature. 

Another reason for using a carrier gas is to suppress the frequency of collisions between the precursor radicals upon plasma condition. The effect is explained in Section 3.2.

**Figure 6:**
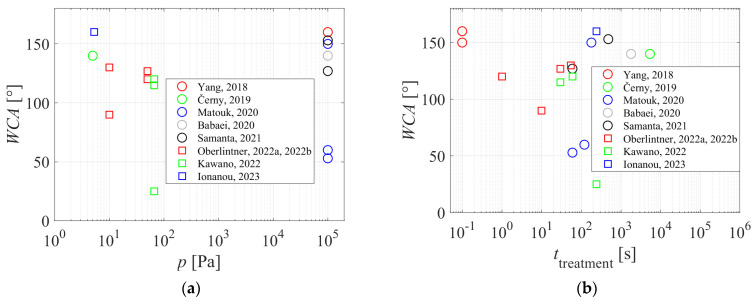
The static water contact angle on cellulose surfaces after deposition of a hydrophobic film by plasma polymerization. (**a**) Versus the pressure, and (**b**) versus the plasma treatment time [16,17,19,22,23,24,25,26,27].

Figure 6a represents the reported WCAs versus the pressure during plasma deposition of hydrophobic coatings. The lack of correlation indicates that both low-pressure and atmospheric-pressure plasmas provide similar results, so the pressure is not the decisive parameter governing the hydrophobicity of treated cellulose materials. The same applies to the treatment time. Figure 6b shows the WCA versus the reported plasma treatment time. Figure 6b reveals that a similar surface wettability is achievable in a broad range of treatment times between 0.1 s and an hour. The shortest treatment time, which enabled the WCA of 150–160°, was reported for plasma jet, which is spatially limited and thus suitable for the treatment of products like foils, textiles, and membranes, and not for three-dimensional objects, let alone powder materials such as nanocellulose.

### 3.2. The Influence of Gas-Phase Reactions

Hydrophobization of cellulose materials by plasma polymerization using HMDSO or similar precursors is illustrated in Figure 2 and Figure 3. The precursor molecules are partially dissociated by electron impact. The resultant radicals diffuse in the gas phase and eventually reach the surface where they condense, thus representing building blocks for a hydrophobic coating. This effect is predominant at low pressures. 

The radicals in the gas phase also collide with each other. If the collision frequency is large so that numerous collisions occur in the gas phase, some radicals will stick to each other in the gas phase. Numerous collisions will cause oligomerization of the radicals in the gas phase, and the oligomers may form clusters. This effect is predominant at atmospheric pressure. Any cluster in the gas phase will charge negatively against plasma due to the attachment of slow plasma electrons.

The negatively charged particles will levitate in the gas phase following the Brownian motion and will not reach the surfaces of the plasma reactor or the substrates because the surfaces facing plasma are also charged negatively. Instead, the negatively charged clusters will serve as gas phase condensation objects for radicals and will grow with time and finally cause the formation of dusty plasma [37]. The dust particles will diffuse in the plasma until reaching the sheath next to the surface, where they will feel the drag forces caused by positively charged ions, which accelerate toward the surface in the voltage drop across the sheath. Finally, the dust particles will adhere to the surfaces and represent the building blocks for a hydrophobic layer. 

The formation of nanoparticles in the gas phase is unavoidable at atmospheric pressure plasma sustained by continuous discharges because of a considerable collision frequency and very short mean free path of gaseous molecules compared to the geometrical dimensions of a plasma reactor. It may also occur in low-pressure plasmas, especially when plasma is sustained by capacitively coupled RF discharge [38]. The nanoparticles were explicitly reported by some authors who recently used atmospheric pressure plasmas for the hydrophobization of cellulose materials [16,17]. The kinetics of nanoparticle formation in the gas phase upon plasma condition is still a subject of scientific research [39]. However, the size of the nanoparticles increases with increasing pressure and plasma duration. The effect is suppressed by pulsed discharges or pulsed injections of the precursor [40]. The nanoparticles deposited from dusty plasma may not adhere well to the cellulose substrates due to the lack of adhesion forces, as explained by Cerny et al. [17]. On the other hand, the rich morphology on the sub-micrometer scale is a necessary condition for super-hydrophobicity. Namely, smooth polymers do not exhibit water contact angles above 130° [41]. Dusty plasma sustained at elevated pressures in HMDSO or similar precursors will, therefore, assure a rich morphology of the hydrophobic coating and thus enable the super-hydrophobic surface finish regardless of the morphology of virgin samples. The adhesion force of the nanoparticles on the cellulose substrate may be questionable, though.

### 3.3. Adhesion of a Hydrophobic Coating and Pre-Treatment with Oxygen Plasma

As early as 1994, Parker [42] stressed the importance of the adhesion of polymer films synthesized by plasma polymerization using HMDSO as a precursor on polymeric substrates. Later, numerous authors used various plasma pre-treatments to improve coating adhesion on the cellulose substrate. The industrial reactors for the deposition of PDMSO-like coatings always employ a pre-treatment with a plasma sustained in oxygen, air, and/or argon [43]. The VUV radiation from gaseous plasma will break bonds in the surface film of the polymer and cause increased surface energy, which is beneficial for the initial adhesion of precursor radicals [44]. Furthermore, chemically reactive species with a high oxidation potential, like O and OH radicals, will interact with surface impurities and remove traces of organic impurities by oxidation and formation of molecules such as CO_2_ and H_2_O. The pre-treatment of cellulose samples by plasma sustained in oxygen, air, or moist argon was reported by all authors who employed HMDSO for the deposition of hydrophobic coatings, as indicated inTable 1. The pre-treatment was also found helpful for the deposition of hydrophobic coatings by standard CVD reactions, i.e., without using plasma conditions [20,21]. The surface oxidation could be performed simultaneously with plasma polymerization using a mixture of oxygen and HMDSO [16]. Still, the surface properties of the deposited films will deviate significantly from polydimethylsiloxane because of extensive oxidation, which occurs during the growth of the hydrophobic film when mixing HMDSO with oxygen [45].

The pre-treatment of cellulose materials with plasma seems unnecessary when using fluorocarbons for cellulose hydrophobization, as indicated in Table 2. The reason is that fluorine atoms formed upon dissociation of fluorocarbon precursors exhibit a high oxidation potential. The interaction of plasma sustained in fluorocarbon gases will cause both the etching and deposition of Teflon-like coatings. The etching of cellulose (which is rich in oxygen) will result in the formation of volatile molecules containing carbon, fluorine, and oxygen [46]. Known molecules include oxy (x = 1) and peroxy (x = 2) radicals of formulae CF_3_O_x_, FC(O)O_x_, CF_3_C(O)O_x_, and CF_3_OC(O)O_x_. Such moieties are implicated in the depletion of the ozone layer, so their formation should be suppressed. Other molecules likely to be synthesized upon the interaction of F atoms with cellulose materials are hydrofluoroethers [47]. The etching is suppressed by the addition of hydrogen or a hydrocarbon gas to the fluorine-containing precursor. The H atoms act as scavengers for F atoms because the heterogeneous surface association leads to the formation of HF molecules. 

### 3.4. The Influence of Water Vapor and Other Gaseous Impurities

The etching of cellulose materials upon treatment with a plasma sustained in CF_4_ is enhanced due to the presence of water vapor in the low-pressure plasma reactor. When the partial pressure of water vapor is significant compared to the partial pressure of tetrafluoromethane, the etching prevails [11]. The optical emission spectroscopy of CF_4_ plasma loaded with cotton textiles revealed extensive etching because the CO bands were by far more intensive than the CF_x_ continua [11]. Unfortunately, the recent authors who reported hydrophobization of cellulose materials by fluorocarbon plasma (Table 2) did not report the partial pressure of water vapor during plasma treatment. Only Oberlintner et al. [25] mentioned the ultimate pressure achieved after evacuating the plasma reactor loaded with cellulose-containing composite. Water vapor is the most abundant gas in hermetically tight vacuum systems operating at room temperature. The cellulose materials are often hygroscopic, so they will likely release water upon vacuum conditions. Namely, the saturated water pressure at room temperature is several 1000 Pa, so it is likely to desorb from cellulose evacuated to the range of pressures used in reviewed articles, i.e., 5–67 Pa (Figure 2 and Table 2).

The desorption of water vapor from cellulose (or any other material) increases with increasing temperature, and the sample temperature increases with increasing plasma treatment time. Namely, all surface reactions upon treatment of solid materials with non-equilibrium gaseous plasma are exothermic, so a cellulose sample will likely be heated upon plasma treatment. The list of highly exothermic surface reactions includes the weak bombardment by positively charged ions, which accelerate in the sheath between the unperturbed plasma and the sample, the neutralization of charged particles, the oxidation of cellulose materials upon chemical interaction with radicals of high oxidation potential (F, O, OH), and surface association of atoms to parent molecules (often referred as heterogeneous surface recombination [48]). Unfortunately, the authors of the review paper did not report the temperature of cellulose samples upon treatment with gaseous plasma. Extensive desorption of water may explain some non-expected results, for example, the highly hydrophilic surface finish obtained after prolonged treatment with CF_4_ plasma. Namely, Kawano et al. [26] reported optimal hydrophobicity (WCA 120°) after treating a cellulose film for a minute, but the WCA dropped to as low as 25° after treating the same material for 4 min.

On the contrary, the addition of hydrogen or hydrocarbons to plasma sustained in fluorinated precursors will suppress the etching of cellulose materials and favor the deposition of hydrophobic films, as reported in the classical paper by D’Agostino et al. [49], or Oehrline et al. [29]. The plasma sustained in pure hydrocarbons may also lead to the hydrophobic surface finish of cellulose materials [19], but the hydrophobicity is inferior to that obtained by plasma treatment in fluorine or silicon-containing gases.

### 3.5. Nanostructuring and Hydrophobization

The etching of polymer materials with gaseous plasma often leads to rich morphology on the sub-micrometer scale. Numerous authors reported nanostructured surfaces after treating a polymer sample with plasmas sustained in different gases by various discharges. Recently, Ioannou et al. [27] reported the WCA of cellulose acetate membranes of 160°. The recipe is as follows—The samples are first treated with a plasma, which causes etching. The etching causes very rich morphology and often a superhydrophilic surface finish. In the next step, the etched samples are coated with a thin hydrophobic film by plasma polymerization. This recipe never fails because the etching and the deposition steps are separated. It is highly recommended for obtaining the super-hydrophobic surface finish of two-dimensional objects like foils and membranes. Namely, such thin electrically non-conductive samples can be exposed to energetic positively charged ions by biasing the surface highly negatively against the plasma. This is performed by placing the thin cellulose material onto the powered electrode of a capacitively coupled discharge sustained at low pressure. The technique is known as reactive ion etching and is widely used in microelectronics for etching of photoresists [50].

### 3.6. Scientific Considerations and Roadmap

The deposition rate of any coating depends on the flux of radicals capable of serving as building blocks of the hydrophobic film and the deposition time. From this perspective, the hydrophobicity should increase with the increasing treatment time. Such behavior was reported by some authors [19,23,24,25], but Figure 2 reveals little correlation between the treatment time and the reported WCA. The paradox is explained by the fact that the WCA is independent of the thickness of the hydrophobic film once the surface is uniformly covered. Also, the substrate roughness on the sub-micrometer scale influences the hydrophobicity significantly.

The flux of radicals or the thickness of the deposited hydrophobic film was not reported in the reviewed articles. Scientists rarely report these parameters. Still, Gosar et al. [48] thoroughly characterized HMDSO plasma in an industrial reactor and estimated the dissociation fraction of precursor molecules to about 10%. In another study, the same group reported the deposition rates at various conditions, including the position of samples in the large plasma reactor [10]. The deposition rates in an industrial-size reactor were between 1 and 15 nm/min. The deposition rates in smaller reactors where plasma is sustained at a relatively large power density may approach 100 nm/min [51]. Supposing that a uniform coating on a smooth surface represents a 1 nm thick coating, the theoretical limit of treatment time will be roughly a second.

The WCA also depends on the surface roughness. Some authors measured it and reported various results. The surface roughness depends on the type of cellulose materials and the etching kinetics. Since the reviewed articles reported various materials and etching procedures, the reported results are not comparable.

Taking into account the upper considerations, there is a need for a more systematic investigation of the hydrophobization of cellulose materials. Smooth samples like cellulose foils should be used to determine the dose (flux times treatment time as long as the flux is constant) of radicals suitable for saturation of the WCA at the level typical for smooth hydrophobic polymers like polytetrafluoroethylene or polydimethylsiloxane. The doses will represent the directions for the calculation of the required treatment times for cellulose samples of more complex geometry. The flux of radicals could be evaluated by mass spectrometry.

The hydrophobization of powder cellulose remains a scientific as well as a technological challenge. Cerny et al. [18] reported encouraging results on the hydrophobization of cellulose fibers in low-pressure plasma sustained in HMDSO. The activation of the fibers by pre-treatment with oxygen plasma was found absolutely necessary for the desired surface finish. The treatment time, however, needed to be shorter to make the methods useful for mass application. Namely, about 100 g of cellulose fibers were stirred in a dish and placed into the plasma reactor, and the required treatment time was about an hour. The result is not surprising if one considers the required treatment times reported by other authors who used foils, membranes, and textiles. Namely, the surface of three-dimensional samples like fibers and powders increases with the square of the linear dimension. 

Finally, adding F-scavengers to gaseous plasma is recommended when the gaseous precursors are fluorocarbons, and rapid hydrophobization is the goal. The effect has been known for decades [29], but none of the recent authors employed it in order to increase the growth of Teflon-like films on cellulose samples. 

## 4. Conclusions

Recent literature on the hydrophobization of cellulose materials by treatment with gaseous plasma was reviewed, and correlations between the degree of hydrophobization as deduced from measured static water contact angles and processing parameters were drawn. Despite significant efforts, knowledge in this niche is still very limited. The exact mechanisms governing the hydrophobization kinetics are not well explained. In particular, there is a lack of information on the fluxes of reactive plasma species onto the cellulose materials. Almost all authors focused on the deposition of a hydrophobic coating using HMDSO or fluorinated carbon precursors. Both low-pressure and atmospheric-pressure plasmas were probed, but no significant difference in the evolution of the cellulose wettability is noticeable from the reviewed literature. Still, plasma methods remain promising for the hydrophobization of cellulose materials because of the low precursor consumption and because they are regarded ecologically acceptable when using fluorine-free precursors. Finally, it should be mentioned that plasma polymerization is one of many methods for achieving highly hydrophobic cellulose materials. A brief review of other methods for attaining the super-hydrophobic surface finish of cellulose paper was published recently [52].

## Figures and Tables

**Table 1:** Summary of methods and results used to deposit PDMSO-like coatings.

Author	Ref.	Pressure	Power [W]	Carrier Gas	Precursor	*t*_treatment_	WCA [°]	Material Type
Yang	[16]	1 bar	N/A	Oxygen	HMDSO	0.1 s	160	Textile
Yang	[16]	1 bar	N/A	Nitrogen	HMDSO	0.1 s	150	Textile
Černy	[17]	1 bar	750	Air	HMDSO	30 min	140	Textile
Černy	[17]	5 Pa	500	None	HMDSO	90 min	140	Powder
Matouk	[19]	1 bar	N/A	Argon	SiH_4_	1 min	53	Film
Matouk	[19]	1 bar	N/A	Argon	SiH_4_	2 min	60	Film
Matouk	[19]	1 bar	N/A	Argon	SiH_4_	3 min	150	Film
Leal	[20]	Plasma pre-treatment only			TCMS		133	Membrane
Yao	[21]	Plasma pre-treatment only			TS-POSS		153	Film
Babaei	[22]	1 bar	N/A	Helium	HMDSO	30 min	140	Paper
Babaei	[22]	1 bar	N/A	Helium	HMDSO	30 min	140	Paper

**Table 2:** Summary of methods and results used to deposit PTFE-like coatings.

Author	Ref.	Pressure	Power [W]	Carrier Gas	Precursor	*t*_treatment_	WCA [°]	Material Type
Samanta	[23]	1 bar	N/A	Helium	C_2_F_4_	1 min	127	Textile
Samanta	[23]	1 bar	N/A	Helium	C_2_F_4_	8 min	153	Textile
Oberlintner	[24]	A few 10 Pa	80	None	CF_4_	10 s	90	Foil
Oberlintner	[24]	A few 10 Pa	80	None	CF_4_	55 s	130	Foil
Oberlintner	[25]	50 Pa	150	None	CF_4_	1 s	120	Composite
Oberlintner	[25]	50 Pa	150	None	CF_4_	30 s	127	Composite
Kawano	[26]	67 Pa	40	None	CF_4_	30 s	115	Film
Kawano	[26]	67 Pa	40	None	CF_4_	60 s	120	Film
Kawano	[26]	67 Pa	40	None	CF_4_	240 s	25	Film
Ionanou	[27]	5.33 Pa	900	None	C_4_F_8_	240 s	160	Membrane

## Data Availability

No new data were created in this study. Data sharing is not applicable to this article.

## References

[1] Karmanov A.P., Kanarsky A.V., Kocheva L.S., Belyy V.A., Semenov E.I., Rachkova N.G., Bogdanovich N.I., Pokryshkin S.A. (2021). Chemical structure and polymer properties of wheat and cabbage lignins—Valuable biopolymers for biomedical applications. Polymer.

[2] Kocheva L.S., Karmanov A.P., Mironov M.V., Belyy V.A., Polina I.N., Pokryshkin S.A. (2020). Characteristics of chemical structure of lignin biopolymer from Araucaria relict plant. Questions and answers of evolution. Int. J. Biol. Macromol..

[3] Oberlintner A., Likozar B., Novak U. (2021). Hydrophobic functionalization reactions of structured cellulose nanomaterials: Mechanisms, kinetics and in silico multi-scale models. Carbohydr. Polym..

[4] Sundriyal P., Pandey M., Bhattacharya S. (2020). Plasma-assisted surface alteration of industrial polymers for improved adhesive bonding. Int. J. Adhes. Adhes..

[5] Luque-Agudo V., Hierro-Oliva M., Gallardo-Moreno A.M., González-Martín M.L. (2021). Effect of plasma treatment on the surface properties of polylactic acid films. Polym. Test..

[6] Ma C., Wang L., Nikiforov A., Onyshchenko Y., Cools P., Ostrikov K., De Geyter N., Morent R. (2021). Atmospheric-pressure plasma assisted engineering of polymer surfaces: From high hydrophobicity to superhydrophilicity. Appl. Surf. Sci..

[7] Hori M. (2022). Radical-controlled plasma processes. Rev. Mod. Plasma Phys..

[8] Liu F., Nie L., Lu X., Stephens J., Ostrikov K. (2020). Atmospheric plasma VUV photon emission. Plasma Sources Sci. Technol..

[9] Lojen D., Zaplotnik R., Primc G., Mozetič M., Vesel A. (2020). Effect of VUV radiation and reactive hydrogen atoms on depletion of fluorine from polytetrafluoroethylene surface. Appl. Surf. Sci..

[10] Gosar Ž., Đonlagić D., Pevec S., Gergič B., Mozetič M., Primc G., Vesel A., Zaplotnik R. (2021). Distribution of the Deposition Rates in an Industrial-Size PECVD Reactor Using HMDSO Precursor. Coatings.

[11] Gorjanc M., Jazbec K., Šala M., Zaplotnik R., Vesel A., Mozetič M. (2014). Creating cellulose fibres with excellent UV protective properties using moist CF_4_ plasma and ZnO nanoparticles. Cellulose.

[12] Fukunaga Y., Longo R.C., Ventzek P.L.G., Lane B., Ranjan A., Hwang G.S., Hartmann G., Tsutsumi T., Ishikawa K., Kondo H. (2020). Interaction of oxygen with polystyrene and polyethylene polymer films: A mechanistic study. J. Appl. Phys..

[13] Vesel A., Zaplotnik R., Mozetič M., Primc G. (2021). Surface modification of PS polymer by oxygen-atom treatment from remote plasma: Initial kinetics of functional groups formation. Appl. Surf. Sci..

[14] Levchenko I., Xu S., Baranov O., Bazaka O., Ivanova E.P., Bazaka K. (2021). Plasma and Polymers: Recent Progress and Trends. Molecules.

[15] Miranda I., Souza A., Sousa P., Ribeiro J., Castanheira E.M.S., Lima R., Minas G. (2021). Properties and Applications of PDMS for Biomedical Engineering: A Review. J. Funct. Biomater..

[16] Yang J., Pu Y., Miao D., Ning X. (2018). Fabrication of Durably Superhydrophobic Cotton Fabrics by Atmospheric Pressure Plasma Treatment with a Siloxane Precursor. Polymers.

[17] Cerny P., Bartos P., Olsan P., Spatenka P. (2019). Hydrophobization of cotton fabric by Gliding Arc plasma discharge. Curr. Appl. Phys..

[18] Cerny P., Bartos P., Kriz P., Olsan P., Spatenka P. (2021). Highly Hydrophobic Organosilane-Functionalized Cellulose: A Promising Filler for Thermoplastic Composites. Materials.

[19] Matouk Z., Torriss B., Rincón R., Dorris A., Beck S., Berry R.M., Chaker M. (2020). Functionalization of cellulose nanocrystal films using Non-Thermal atmospheric—Pressure plasmas. Appl. Surf. Sci..

[20] Leal S., Cristelo C., Silvestre S., Fortunato E., Sousa A., Alves A., Correia D.M., Lanceros-Mendez S., Gama M. (2020). Hydrophobic modification of bacterial cellulose using oxygen plasma treatment and chemical vapor deposition. Cellulose.

[21] Yao M.Z., Liu Y., Qin C.N., Meng X.J., Cheng B.X., Zhao H., Wang S.F., Huang Z.Q. (2021). Facile fabrication of hydrophobic cellulose-based organic/inorganic nanomaterial modified with POSS by plasma treatment. Carbohydr. Polym..

[22] Babaei S., Profili J., Asadollahi S., Sarkassian A., Dorris A., Beck S., Stafford L. (2020). Analysis of transport phenomena during plasma deposition of hydrophobic coatings on porous cellulosic substrates in plane-to-plane dielectric barrier discharges at atmospheric pressure. Plasma Process. Polym..

[23] Samanta K.K., Joshi A.G., Jassal M., Agrawal A.K. (2021). Hydrophobic functionalization of cellulosic substrate by tetrafluoroethane dielectric barrier discharge plasma at atmospheric pressure. Carbohydr. Polym..

[24] Oberlintner A., Shvalya V., Vasudevan A., Vengust D., Likozar B., Cvelbar U., Novak U. (2022). Hydrophilic to hydrophobic: Ultrafast conversion of cellulose nanofibrils by cold plasma fluorination. Appl. Surf. Sci..

[25] Oberlintner A., Vesel A., Naumoska K., Likozar B., Novak U. (2022). Permanent hydrophobic coating of chitosan/cellulose nanocrystals composite film by cold plasma processing. Appl. Surf. Sci..

[26] Kawano T., Wang M.J., Andou Y. (2022). Surface Modification of a Regenerated Cellulose Film Using Low-Pressure Plasma Treatment with Various Reactive Gases. ACS Omega.

[27] Ioannou D., Hou Y., Shah P., Ellinas K., Kappl M., Sapalidis A., Constantoudis V., Butt H.J., Gogolides E. (2023). Plasma-Induced Superhydrophobicity as a Green Technology for Enhanced Air Gap Membrane Distillation. ACS Appl. Mater. Interfaces.

[28] Booth J.-P. (1999). Optical and electrical diagnostics of fluorocarbon plasma etching processes. Plasma Sources Sci. Technol..

[29] Oehrlein G.S., Williams H.L. (1987). Silicon etching mechanisms in a CF_4_/H_2_ glow discharge. J. Appl. Phys..

[30] Zanini S., Riccardi C., Orlandi M., Fornara V., Colombini M.P., Donato D.I., Legnaioli S., Palleschi V. (2007). Wood coated with plasma-polymer for water repellence. Wood Sci. Technol..

[31] Mahlberg R., Niemi H.E.M., Denes F., Rowell R.M. (1998). Effect of oxygen and hexamethyldisiloxane plasma on morphology, wettability and adhesion properties of polypropylene and lignocellulosics. Int. J. Adhes. Adhes..

[32] Inagaki N., Tasaka S., Mori K. (1991). Hydrophobic polymer films plasma-polymerized from CF_4_/hydrocarbon and hexafluroacetone/hydrocarbon mixtures. J. Appl. Polym. Sci..

[33] Hochart F., Levalois-Mitjaville J., De Jaeger R., Gengembre L., Grimblot J. (1999). Plasma surface treatment of poly(acrylonitrile) films by fluorocarbon compounds. Appl. Surf. Sci..

[34] Benedikt J. (2010). Plasma-chemical reactions: Low pressure acetylene plasmas. J. Phys. D Appl. Phys..

[35] Dorai R., Kushner M.J. (2003). A model for plasma modification of polypropylene using atmospheric pressure discharges. J. Phys. D Appl. Phys..

[36] National Institute of Standards and Technology NIST Chemistry WebBook, SRD 69. https://webbook.nist.gov/cgi/cbook.cgi?ID=C107460&Units=SI&Mask=4&Type=ANTOINE&Plot=on.

[37] Despax B., Makasheva K., Caquineau H. (2012). Cyclic powder formation during pulsed injection of hexamethyldisiloxane in an axially asymmetric radiofrequency argon discharge. J. Appl. Phys..

[38] Garofano V., Bérard R., Glad X., Joblin C., Makasheva K., Stafford L. (2019). Time-resolved analysis of the precursor fragmentation kinetics in an hybrid PVD/PECVD dusty plasma with pulsed injection of HMDSO. Plasma Process. Polym..

[39] Popok V.N., Kylián O. (2021). Formation of Advanced Nanomaterials by Gas-Phase Aggregation. Appl. Nano.

[40] Cacot L., Carnide G., Kahn M.L., Clergereaux R., Naudé N., Stafford L. (2022). Kinetics driving thin-film deposition in dielectric barrier discharges using a direct liquid injector operated in a pulsed regime. J. Phys. D Appl. Phys..

[41] Diversified Enterprises Critical Surface Tension and Contact Angle with Water for Various Polymers. https://www.accudynetest.com/polytable_03.html?sortby=contact_angle.

[42] Parker J.L., Claesson P.M., Wang J.-H., Yasuda H.K. (2002). Surface Forces between Plasma Polymer Films. Langmuir.

[43] Fernandes J.C.S., Ferreira M.G.S., Haddow D.B., Goruppa A., Short R., Dixon D.G. (2002). Plasma-polymerised coatings used as pre-treatment for aluminium alloys. Surf. Coat. Technol..

[44] Zhang Y., Ishikawa K., Mozetič M., Tsutsumi T., Kondo H., Sekine M., Hori M. (2019). Polyethylene terephthalate (PET) surface modification by VUV and neutral active species in remote oxygen or hydrogen plasmas. Plasma Process. Polym..

[45] Chaiwong C., Rachtanapun P., Sarapirom S., Boonyawan D. (2013). Plasma polymerization of hexamethyldisiloxane: Investigation of the effect of carrier gas related to the film properties. Surf. Coat. Technol..

[46] Von Ahsen S., Willner H., Argüello G.A. (2004). Fluorocarbon oxy and peroxy radicals. J. Fluor. Chem..

[47] Saathoff H., Zellner R. (1993). LIF detection of the CF_3_O radical and kinetics of its reactions with CH_4_ and C_2_H_6_. Chem. Phys. Lett..

[48] Paul D., Mozetic M., Zaplotnik R., Primc G., Donlagic D., Vesel A. (2023). A Review of Recombination Coefficients of Neutral Oxygen Atoms for Various Materials. Materials.

[49] D’Agostino R., Cramarossa F., Illuzzi F. (1987). Mechanisms of deposition and etching of thin films of plasma-polymerized fluorinated monomers in radio frequency discharges fed with C_2_F_6_-H_2_ and C_2_F_6_-O_2_ mixtures. J. Appl. Phys..

[50] Huff M. (2021). Recent Advances in Reactive Ion Etching and Applications of High-Aspect-Ratio Microfabrication. Micromachines.

[51] Petit-Etienne C., Tatoulian M., Mabille I., Sutter E., Arefi-Khonsari F. (2007). Deposition of SiOχ-Like Thin Films from a Mixture of HMDSO and Oxygen by Low Pressure and DBD Discharges to Improve the Corrosion Behaviour of Steel. Plasma Process. Polym..

[52] Yun T., Tao Y., Li Q., Cheng Y., Lu J., Lv Y., Du J., Wang H. (2023). Superhydrophobic modification of cellulosic paper-based materials: Fabrication, properties, and versatile applications. Carbohydr. Polym..

